# Retrospective evaluation of seven different treatment protocols in hospitalized COVID-19 patients

**DOI:** 10.3906/sag-2106-114

**Published:** 2021-08-20

**Authors:** Kübra DEMİR ÖNDER, Ayşegül SEREMET KESKİN, Hande BERK, Derya SEYMAN, Nefise ÖZTOPRAK

**Affiliations:** Department of Infectious Diseases and Clinical Microbiology, University of Health Sciences Antalya Training and Research Hospital, Antalya, Turkey

**Keywords:** COVID-19, treatment, favipiravir, remdesivir, hydroxychloroquine

## Abstract

**Background/aim:**

As the experience has increased regarding SARS-CoV-2 in time, treatment trends have changed since the beginning of the pandemic. This study aimed to compare the outcomes of different treatment modalities for inpatients in a tertiary pandemic hospital in Antalya, Turkey.

**Materials and methods:**

Individuals aged 18 years and above who tested positive for SARS-CoV-2 in PCR with presenting COVID-related radiological findings, hospitalized for at least 3 days, and completed follow-up between March 15, 2020 and November 30, 2020 were included in the study. Patients’ data were reviewed retrospectively. Seven treatment groups based on the single or combined use of hydroxychloroquine, oseltamivir, favipiravir, and remdesivir were formed and compared in terms of mortality, survival, length of hospital stay, need for intensive care, and mechanical ventilation.

**Results:**

A total of 321 patients were included in the study. The length of hospital stay, the need for intensive care, and mechanical ventilation were lower in Group 1 (hydroxychloroquine) and Group 2 (hydroxychloroquine + oseltamivir) compared to the other groups (p < 0.05). No significant difference was determined in survival between treatment groups. Analysis of prognostic factors affecting overall survival revealed that the need for intensive care and mechanical ventilation increased mortality [11.1 times (p < 0.001) and 6.48 times (p < 0.001), respectively].

**Conclusion:**

No significant difference was determined between different treatment protocols in terms of their impact on survival. To end the COVID-19 pandemic, there is an urgent need to develop highly efficient, rapid-acting, and orally available antiviral drugs.

## 1. Introduction

The SARS-CoV-2 pandemic continues to exist across the world. Many agents currently used in treating COVID-19 are drugs such as hydroxychloroquine (HCQ), oseltamivir, favipiravir (FAV), and remdesivir, which have been effective in previous pandemics [1].

Since December 2019, when COVID-19 was identified for the first time, treatment protocols in Turkey have changed over time based on global and local guide recommendations and changes in COVID-19 treatment trends. In March 2020, the first case was determined in Turkey, and treatment regimens according to the guidelines of the Ministry of Health on COVID-19 began to be applied in COVID-19 treatment^1^. Moreover, COVID-19 drugs are provided free of charge by the Ministry of Health. At the beginning of the pandemic, only lopinavir/ritonavir and HCQ were available for COVID-19 treatment in Turkey. A few months later, FAV could be imported and administered alone or combined with HCQ to patients at a treatment dose of only 5 days. Towards the end of August 2020, FAV could be prescribed for 10 days.

Physicians from different countries used remdesivir, donated by the manufacturer, in COVID-19 disease (“compassionate use”) [2]. Remdesivir, which was available in limited numbers in our hospital, was obtained by applying to the Republic of Turkey, Ministry of Health Drugs and Medical Devices Agency and could only be used in a few patients who were unresponsive to initial treatment and had a severe clinical condition.

Initially, steroids were not recommended for the treatment of COVID-19. However, they began to be used after the inclusion of “6 mg/day of dexamethasone, 40 mg/day of prednisolone or 32 mg/day of methylprednisolone can be prescribed for patients who required oxygen” in the COVID-19 treatment guidelines of the Ministry of Health on August 2, 2020^2^. Tocilizumab is in the “off-label” treatment category for COVID-19 in Turkey and is used in patients with cytokine storms upon application to the health authority and the subsequent approval. High-cost “cytokine adsorption” (via cytokine adsorber, CytoSorb ^®^) can only be administered to patients who are admitted to intensive care, do not respond to standard treatment and steroids, and have a severe disease and cytokine storm.

This study aimed to retrospectively evaluate the effects of four primary drugs (HCQ, oseltamivir, FAV, and remdesivir) and seven different treatment protocols consisting of a combination of these drugs on hospitalization, need for intensive care and mechanical ventilation, and survival in patients who met the inclusion criteria in our tertiary training and research hospital assigned as a pandemic hospital since the first case was identified in Turkey.

## 2. Materials and methods

The study retrospectively included 321 patients aged 18 and above who tested positive for SARS-CoV-2 in PCR test with COVID-related pneumonia in the computerized tomography of the chest and were hospitalized for at least 3 days with a completed follow-up between March 15, 2020 and November 30, 2020. Even if their tomography findings were compatible with COVID-19 pneumonia, cases not confirmed by a PCR test, pregnant women, and patients under 18 years of age were excluded.

Seven main treatment groups were defined. Since HCQ + azithromycin and lopinavir/ritonavir were used for a short period in very few patients, those who received these treatments were not included in the study. The main treatment groups in this study were as follows: Group 1 = HCQ for 5 days, Group 2 = HCQ + oseltamivir for 5 days, Group 3 = FAV for 5 days, Group 4 = FAV for 5 days after HCQ ± oseltamivir for 5 days, Group 5 = FAV for 10 days, Group 6 = FAV + HCQ for at least 5 days, Group 7 = Remdesivir treatment after FAV for at least 5 days.

In addition, whether the treatments of immune plasma (one dose of 200 mL), cytokine adsorption (CytoSorb^®^, for 3 days), low molecular weight heparin (LMWH, enoxaparin sodium 4000 or 6000 IU subcutaneously daily), vitamin C (2000 mg intravenously daily), and tocilizumab (400 mg or 800 mg/total dose) were administered in each of these groups was recorded. The main treatment groups were also compared regarding patients’ length of hospital stay, need for intensive care and mechanical ventilation, and mortality.

The severity of pneumonia was classified at the initiation of treatment based on the low-dose unenhanced chest tomography results^3^.

Unilateral or bilateral, peripheral or adjacent to fissure, small, partially round ground-glass opacities, mainly in the lower lobes, were staged as “mild”; bilateral, multifocal, more extended involvements in tomography as less than 50% of the parenchyma, up to the upper lobes of the lung, were staged as “moderate”; widespread involvement over 50% of the bilateral lung parenchyma, consolidation accompanying ground-glass opacities, or tomography findings of air bronchogram or crazy-paving were staged as “severe” pneumonia [3].

Standard treatment doses in each patient were as follows: 2 × 200 mg of HCQ (orally) for 5 days, 2 × 75 mg of oseltamivir for 5 days, 2 × 1600 mg of FAV on day zero and 2 × 600 mg on other 4 or 9 days, and 200 mg of remdesivir (intravenously) on the first day and 100 mg/ day for 4 days.

### 2.1. Statistical analysis

The data were analyzed using IBM SPSS Statistics 18, 2010 software. The Kolmogorov–Smirnov test determined the compliance of continuous variables to normal distribution. The categorical variables were presented in frequency and percentage, and continuous variables in mean, standard deviation, median, minimum, and maximum values. The chi-square significance test analyzed the categorical variables and made a post hoc Bonferroni correction. One-way ANOVA determined the mean value comparisons of more than two groups, post hoc LSD tests were used when parametric test assumptions were met, and the Kruskal–Wallis and post hoc pairwise comparison tests in other cases. The survival rates were estimated using the Kaplan–Meier method, and a log-rank test was performed to examine whether there was a difference between the variable levels in terms of survival probabilities. Next, Cox regression analysis determined the factors affecting survival. Possible factors identified by univariate analyses were analyzed with a multiple logistic regression model. The statistical significance level was considered to be 0.05 in the study.

## 3. Results

### 3.1. General demographic and clinical characteristics

Three hundred twenty-one patients who met the inclusion criteria were included in the study. One hundred eighty-nine patients (58.9%) were male, and the mean age was 56.11 ± 14.67 years. Of the 274 patients with an accessible smoking history, 87 (31.8%) were smokers. The mean time from the onset of symptoms to the initiation of the main treatment was 3.57 ± 3.41 days. One hundred two patients (31.8%) had mild COVID-19 pneumonia, whereas 116 (36.1%) had severe COVID-19 pneumonia on admission. The mean length of hospital stay of the patients was 12 ± 10.31 days, and the mean length of stay in intensive care was 15.38 ± 11.86 days. Ninety-eight patients (30.5%) required intensive care and 67 (20.9%) mechanical ventilation during follow-up. The main treatment groups and the number of patients in each group were as follows:

**Group 1:** HCQ for 5 days (n = 44) (13.7%)
**Group 2:** HCQ+ oseltamivir for 5 days (n = 31) (9.7%)
**Group 3:** FAV for 5 days (n = 73) (22.7%)
**Group 4:** FAV for 5 days after HCQ ± oseltamivir (n = 33) (10.3%)
**Group 5:** FAV for 10 days (n = 82) (25.5%)
**Group 6:** FAV+HCQ for at least 5 days (n = 41) (12.8%)
**Group 7:** Remdesivir after FAV for at least 5 days (n = 17) (5.3%)

Other than the main treatments, steroid treatment was administered to 124 patients (38.6%) due to low oxygen saturation during the follow-up. Methylprednisolone was the most common steroid type and was administered to 68.5% of patients (85/124). The steroids were added to the treatment regime with an average of 6.19 ± 3.53 days after symptom onset. Fifty-eight (18.1%) patients underwent immune plasma therapy, and the mean value of the time from the symptom onset to the administration of immune plasma was 8.69 ± 4.62 days. LMWH was administered in 266 patients (82.9%) after an average of 4.91 ± 3.60 days from the onset of symptoms. Two hundred forty-four (76%) patients were applied intravenous vitamin C of 2 g/day during their stay at the hospital. Tocilizumab was applied to 15 patients (4.7%) diagnosed with cytokine storm associated with COVID-19 and cytokine adsorption to 22 patients (6.9%), and both of them to 5 patients. The overall mortality rate due to all causes was 20.6% (n = 66) with the following reasons: cardiac arrest (n = 35), sepsis (n = 15 (bacterial sepsis = 13 and fungal sepsis = 2)), multiorgan failure (n = 10) and respiratory arrest (n = 6). The mortality rate within 14 days after diagnosis was 7.5% (n = 24). The general demographic and clinical characteristics of the patients are presented in Table 1.

### 3.2. Presenting symptoms and comorbidities

The patients were most frequently presented with symptoms of dry cough (n = 197, 61.4%), fever (n = 180, 56.1%), and fatigue (n = 155, 48.4%). There was at least one comorbidity in 52% of the patients (n = 167). The survival rate was significantly lower in those with comorbidity than those without (p < 0.001). The most common comorbidity was hypertension (n = 104, 32.4%) and patients with hypertension had a lower survival rate than those with other comorbidities (p < 0.001). The second most common comorbidity was diabetes mellitus (n = 91, 28.3%).

### 3.3. Laboratory data for presenting values

Table 1 indicates the general laboratory data on admission and the highest Interleukin-6 (IL-6) and D-Dimer levels during the follow-up.

### 3.4. Demographic and clinical characteristics by main treatment groups

The mean age of the patients in Group 5 was higher than all other groups (p < 0.05). The mean age of the patients in Groups 1 and 2 was lower than those in Groups 3, 5, and 6 (p < 0.05). Group 1 had the lowest mean age (47.20 ± 13.58 years). The mean age values of the groups were similar except for Group 1, 2, and 5. The smoking rate in Group 1 was significantly lower than Group 7 (14.3% and 57.1%, respectively) (p < 0.05). There was no significant difference in smoking rates in the other groups. There was no significant difference between the seven main treatment groups regarding the time from the onset of symptoms to the initiation of treatment. Pneumonia severity at the beginning of treatment was similar in Groups 1 and 2, and these groups had the highest rate of mild pneumonia (p < 0.05). Groups 5, 6, and 7 had a similar and highest rate of severe pneumonia, and Groups 1 and 2 had the lowest rate (p < 0.05). The length of hospitalization in the inpatient clinic was shorter in Group 2 than in Groups 4 and 5 (p < 0.05). There was no significant difference between the other groups in this regard. The total length of hospital stay was shorter in Groups 1 and 2 (p < 0.05) and higher in Group 7 than the others (p < 0.05). The admission rate to intensive care was similar in Groups 1 and 2 and significantly lower than the others (p < 0.05). It was similar in Groups 6 and 7, as the highest in Group 7 (p < 0.05). Mechanical ventilation rate was similar in Groups 1 and 2 and significantly lower than the others (p < 0.05). Groups 3, 4, 5, and 6 had similar results, and Group 7 had the highest rate (p < 0.05). Demographic and clinical characteristics by the treatment groups are summarized in Table 2.

### 3.5. Laboratory data for the presenting values in main treatment groups

The mean value of fasting blood glucose in Group 1 was lower than Groups 3, 5, 6, and 7. The difference was statistically significant (p < 0.05). At the initial admission, C-reactive protein (CRP), albumin level, neutrophil lymphocyte ratio (N/L), lactate dehydrogenase (LDH) level, ferritin level, and peak D-dimer levels were similar in Groups 1 and 2 and lower than the other groups, which was statistically significant (p < 0.05). Lymphocyte count was significantly lower in Group 7 than in Groups 1 and 2 (p < 0.05). Laboratory data for the presenting values of the seven treatment groups are presented in Table 3.

### 3.6. Comorbidities by the groups

The difference between Groups 1 and 5 in terms of comorbidities was statistically significant, and the rate of comorbidities was significantly lower in Group 1 (p < 0.05). There was no significant difference between the other groups in terms of comorbidities. In Group 1, diabetes mellitus (DM) was significantly less common than Groups 3 and 5, and Hypertension (HT) was less common than Groups 5 and 6 (p < 0.05). Comorbidities by treatment groups are shown in Table 4.

### 3.7. Mortality rates by treatment groups

Overall mortality and 28-day mortality were lower in Groups 1 and 2 than the others (p < 0.05). Mortality rates by the treatment groups are summarized in Table 5.

### 3.8. Treatments and practices other than main treatment by the groups

Steroid use was higher in Groups 5, 6, and 7 than others (p < 0.05). It was found that steroids were given to 69.5% of Group 5 (n = 57), 46.3% of Group 6 (n = 19), and 76.5% of Group 7 (n = 13). Immune plasma was administered to 82.4% of Group 7 at a higher rate than the other groups (p < 0.05). The remaining groups had no significant difference in the use of immune plasma. Among the main treatment groups, the LMWH was used least in Group 2 (6.5%), and the difference was statistically significant (p < 0.05). The use of LMWH was 72.7%–100% in the remaining groups, with no significant difference between groups. Vitamin C use was higher in Groups 4, 5, and 7 than in other groups, and the difference was statistically significant (p < 0.05). Tocilizumab was used in only 4.7% (n = 15) of the patients, and 6 of them were in Group 6 (14.8%), and three were in Group 7 (17.6%). The cytokine adsorption rate was higher in Group 7 than the others (p < 0.05). We found that 8 of the 22 patients who underwent cytokine adsorption were in Group 7, and 47.1% of Group 7 had cytokine adsorption. Treatments and practices other than the main treatment by the groups are presented in Table 6.

### 3.9. Results of survival analysis

The mean age of patients who died was significantly higher than those who survived (67.77 ± 10.39 vs 53.09 ± 14.11 years, p < 0.001). The survival rate was examined based on the severity of pneumonia at the beginning of antiviral therapy and found as 94.1% in patients with mild pneumonia and 65% in those with severe pneumonia, and the difference was significant (p < 0.001). The pneumonia was analyzed in two groups as “mild + moderate” and “severe” based on its severity. The survival rate was 87.3% in “mild + moderate” pneumonia and 65.5% in severe pneumonia (p < 0.001). The survival rate was lower in those with comorbidity than those without (71.3% vs. 88.35) (p < 0.001). The survival rate in patients who required intensive care during follow-up was significantly lower than those who did not (34.7% and 99.1%, respectively) (p < 0.001). Among them, the survival rate was much lower in patients who required mechanical ventilation compared to those who did not (13.4% and 96.9%, respectively) (p < 0.001). It was noteworthy that the survival was higher in patients who did not receive immune plasma than in those who did (89% vs. 36.2%) (p < 0.001). As expected, survival was lower in the group of patients in severe condition who underwent tocilizumab and cytokine adsorption (p < 0.001 and p < 0.001, respectively). The survival rate was higher in Group 1 (HCQ for 5 days) and Group 2 (HCQ + oseltamivir for 5 days) compared to the others, and the difference was significant (p < 0.001). Survival rates by the demographic and clinical characteristics are shown in Table 7.

The survival analysis on the general clinical characteristics of the patients through the Kaplan–Meier and log-rank tests revealed that sex, smoking, presence of any comorbidity, and severity of pneumonia at the beginning of treatment did not lead to any statistically significant difference in survival. We found that survival was significantly lower in those admitted to intensive care and those mechanically ventilated, and the difference was statistically significant (p < 0.001 and p < 0.001, respectively). In univariate Cox regression analyses performed to determine the prognostic factors affecting overall survival, we found that intensive care unit (ICU) admission increased mortality by 11.1 times (p < 0.001), and mechanical ventilation increased mortality by 6.48 times (p < 0.001). The variables found significant were included in the multivariate Cox regression analysis. Multivariate Cox regression analysis showed that mechanical ventilation increased mortality by 3.987 times (p < 0.001).

In the multivariate Cox regression analysis, there was no significant difference between the main treatment groups’ impact on survival. Survival analyses by the treatment groups and main clinical characteristics are shown in Table 8. Cumulative survival by the main treatment groups is presented in Figure.

Possible factors identified by univariate analyses were analyzed with a multiple logistic regression model. Logistic regression analysis of risk factors for mortality is shown in Table 9. Accordingly, every 1 year of increase in age increased mortality 1.074 times (p = 0.005); ICU hospitalization increased mortality 14.7 times (p = 0.003), and the need for mechanical ventilation increased mortality 29.1 times (p < 0.001).

## 4. Discussion

At the beginning of the COVID-19 pandemic, there were problems in drug supply in Turkey and the world. In the first month of the pandemic in Turkey, only HCQ could be used in treating hospitalized patients. FAV was included in treatment later on. Remdesivir was available in a limited number and could not be used later due to the depletion of donated medicine stock. Tocilizumab, on the other hand, is used under the control and permission of the health authority. Therefore, treatment protocols had to be organized depending on the drugs we had access to at that moment.

In March 2020, the first COVID-19 case was identified in Turkey. Since then, the scope and duration of the treatments have changed due to the changes in treatment guidelines and the drugs available. We performed this study to clarify which one of the main treatments was superior in survival and found no significant differences between the groups regarding the impact on survival, according to the multivariate Cox regression analysis.

To interpret the results better, we examined the variables such as the number of patients in the groups, their clinical-demographic characteristics, the initiation of treatment, the severity of pneumonia at the beginning of treatment, anticytokines, and supportive therapies in addition to the main antiviral therapies.

Advanced age is the most critical risk factor for mortality in COVID-19 disease [4,5]. We found that the mean age of the patients with mortality was higher, as 67.77 ± 10.39 years.

The most common comorbidities in COVID-19 patients are hypertension, diabetes, and coronary heart disease, respectively. The mechanism of action of hypertension on the course of COVID-19 disease and mortality, in particular, remains unclear [6]. Research by Taylor et al. on risk factors associated with mortality revealed that hypertension is a critical risk factor for mortality [7]. In our study, survival was lower in those with comorbidity than in those with none. The most common comorbidity was hypertension, and patients with hypertension had lower survival than those with other comorbidities.

The severity of pneumonia is directly correlated to the severity of the disease and mortality [8]. In our study, the survival rate was significantly higher in patients with mild pneumonia than those with severe pneumonia (94.1% and 65.5%, respectively) according to the survival rates examined by regression analysis based on the severity of pneumonia at the time of initiation of antiviral therapy. The severity of pneumonia was classified into two groups as “mild + moderate” and “severe”, and it was found that the survival rate in severe pneumonia was significantly lower. We know that early initiation of antiviral therapy in COVID-19 is critical in preventing poor clinical prognosis [5,9]. In our study, the mean value of time from the symptom onset to the main treatment was 3.57 ± 3.41 days. There was no significant difference between the seven treatment groups regarding this period.

In this study, the overall mortality rate was 20.6% (n = 66), and the mortality rate within 14 days after diagnosis was 7.5% (n = 24). A study analyzing the mortality in hospitalized COVID-19 patients reported the overall mortality rate as 25% [10].

Our study determined that admission to the intensive care unit and the need for mechanical ventilation were the most notable factors affecting mortality. Ninety-eight patients (30.5%) were admitted to intensive care during follow-up, and 67 required mechanical ventilation. The average length of stay in intensive care was 15.38 days. Similarly, Wu et al. reported that 29.64% of the patients required intensive care and the average length of stay in the intensive care unit was 18 days [5].

As for the nonantiviral treatments, we found that 124 patients (38.6%) were provided with steroid treatment during the follow-up, and the steroid type given to 85 of these patients was methylprednisolone (68.5%). Dexamethasone was recommended primarily in the Infectious Diseases Society of America (IDSA) guide updated in February 2021. However, our study found no significant difference between steroid type and survival [11].

Immune plasma therapy administered in intubated patients and at a later stage in the disease course may be harmful rather than beneficial due to the already developed antibodies and organ damage associated with the hyper-immune host response^4^ [1].

Our study found that immune plasma was administered to 58 (18.1%) of the patients. It was noteworthy that the survival rate was lower in patients receiving immune plasma. This result was associated with the fact that the immune plasma was administered as salvage therapy to patients requiring hospitalization, a mean of 8.69 ± 4.62 days after the onset of symptoms.

Tocilizumab was administered to 15 patients (4.7%) and cytokine adsorption to 22 patients (6.9%) diagnosed with COVID-19–related cytokine storm. Survival rates were lower in patients who underwent tocilizumab and cytokine adsorption. Recent studies have reported that tocilizumab would be more beneficial if administered early (in the first 48 h) in patients admitted to intensive care for rapidly progressing COVID-19 disease [12]. We found that high-cost cytokine adsorption and tocilizumab, which requires a specific procedure and permission for its procurement, can only be applied to those admitted to intensive care and do not respond to standard therapy and steroids and have advanced stage and severe disease. We think that this might have led to low survival rates in both treatments.

Survival duration was significantly higher in Groups 1 and 2 compared to other groups. We think that this might be due to several factors. First, Groups 1 and 2 had higher rates of mild pneumonia than the others. Groups 5, 6, and 7 had the highest rate of severe pneumonia. Moreover, we know that the disease progresses more severely at an advanced age and in the presence of comorbidities [5]. The mean age in Groups 1 and 2 was lower, and the comorbidity rate was reduced compared to the other groups. Patients in Groups 1 and 2 (HCQ-based groups) stayed in the hospital for a shorter period in total and had lower rates of intensive care and mechanical ventilation. The observational study by Geleris et al. reported that the use of HCQ did not have any significant effect on the need for intubation or mortality [13]. Moreover, since Groups 1 and 2 had more cases of mild pneumonia, and laboratory values for the poor prognosis (lymphocyte count, N/L ratio, LDH level, ferritin level, d dimer level, CRP) were better than the other groups, and the difference was statistically significant.

Studies report that HCQ treatment has no positive effect on clinical improvement and mortality in COVID-19 pneumonia. However, a randomized controlled study has emphasized that when administered to patients with mild pneumonia, a significant difference is found in clinical improvement compared to the control group [14–16]. In Groups 1 and 2, in which the main treatment was HCQ and HCQ + Oseltamivir, mortality and intensive care admission rates were lower since most patients had mild pneumonia.

The survival rate in patients who required intensive care was lower than those who were not (34.7% and 99.1%, respectively). Among these patients, patients who required mechanical ventilation had lower survival rates than those who did not (13.4% and 96.9%, respectively). In terms of prognostic factors affecting overall survival, univariate Cox regression analysis revealed that ICU admission increased mortality by 11.1 times and mechanical ventilation increased mortality by 6.48 times. The variables found significant were included in the multivariate Cox regression analysis. The analysis found that mechanical ventilation increased mortality by 3.9 times. Moreover, some studies have indicated that mechanical ventilation has no significant effect on mortality [7].

### 4.1. Limitations of the study

Our study has some limitations. Firstly, the number of patients was substantially low in the remdesivir group since we were able to reach a limited number of remdesivir, and use it only in patients who did not respond to treatment and had a severe clinical picture. Secondly, we were not able to achieve homogeneity between the groups in terms of the number of patients and the severity of the clinical situation. This was because some treatment protocols were used for a short time and in a small number of patients, but some protocols were used for a longer period of time and a larger number of patients, and the retrospective nature of the study.

### 4.2. Conclusion

The World Health Organization Solidarity study reports that remdesivir, HCQ, lopinavir, and interferon regimens have little or no effect on overall mortality, need for ventilation, and length of hospital stay in COVID-19 patients [17]. Similarly, the IDSA guidelines updated in February 2021 report that no clear positive benefit could be obtained from any treatment option, considering the profit and loss among the COVID-19 treatment options [11].

The study by Ciftciler et al., in which they compiled scientific publications on COVID-19 in Turkey in the first year of the pandemic, reported that most studies were about transmission, prevention, characteristics of the disease, and diagnosis, and very few of them were clinical trials regarding “treatment” [18]. The literature referenced in this study and the last one-year period after this study was searched from the Pubmed database. No other clinical study was found in Turkey in which so many treatment protocols were evaluated together in COVID-19 treatment. Therefore, this study is critical in being a chronological reflection of the different treatment options used in the first year of the pandemic in Turkey.

In our study, which evaluated the seven main treatment protocols, such as “HCQ”, “HCQ+oseltamivir”, “5-day FAV”, “10-day FAV”, “HCQ followed by FAV”, “HCQ+ FAV”, and “FAV followed by remdesivir”, there was no significant difference between the groups in terms of the effect on survival, according to the Multivariate Cox regression analysis.

Mutations that develop in the virus structure have reduced the number of treatment options and vaccine effectiveness. For this reason, the pandemic continues to hit the world without losing its effect. This study revealed that admission to intensive care increased mortality by 11.1 times, and mechanical ventilation increased mortality by 6.48 times. Therefore, apart from the current treatment options for COVID-19, it is believed that we need newer, faster, and more effective antiviral therapies that can be used orally in outpatient treatment without the need for intensive care or mechanical ventilation.

## Figures and Tables

**Figure:**
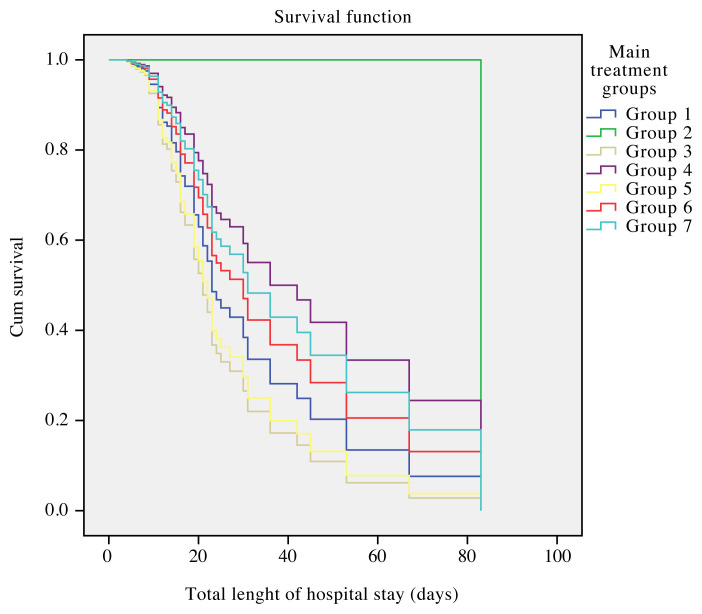
Cumulative survival in main treatment groups.

**Table 1:** General demographic-clinical characteristics and general laboratory data (n = 321).

Age (years)		56.11 ± 14.67 (mean ± SD)	19–91 (56) min–max (med)
Sex (male)		189 (n)	58.9 (%)
Smoker		87/274 (n)	31.8 (%)
Time from the symptom onset to the initiation of main treatment (days)		3.57 ± 3.41 (mean ± SD)	0–24 (3) min–max (med)
Intensive care stay (days)		15.38 ± 11.86 (mean ± SD)	1–82 (12) min–max (med)
Inpatient clinic stay (days)		6.01 ± 5.84 (mean ± SD)	0–29 (4) min–max (med)
Total length of hospital stay (days)		12.00 ± 10.31 (mean ± SD)	3–83 (9) min–max (med)
Need for intensive care		98 (n)	30.5 (%)
Mechanical ventilation		67 (n)	20.9 (%)
Pneumonia severity at the beginning of the main treatment			
Mild		102 (n)	31.8 (%)
Moderate		103 (n)	32.1 (%)
Severe		116 (n)	36.1 (%)
Patients receiving steroids		124 (n)	38.6 (%)
Steroid type (n: 124)			
Methylprednisolone		85 (n)	68.5 (%)
Dexamethasone		39 (n)	31.5 (%)
Time from symptom onset to the administration of steroids (days)		6.19 ± 3.53 (mean ± SD)	0–15 (6) min–max (med)
Patients receiving immune plasma		58 (n)	18.1 (%)
Time from symptom onset to the administration of immune plasma (days)		8.69 ± 4.62 (mean ± SD)	0–23 (8) min–max (med)
Patients receiving LMWH		266 (n)	82.9 (%)
Time from the onset of symptoms to the administration of LMWH (days)		4.91 ± 3.60 (mean ± SD)	0–21 (4) min–max (med)
Patients receiving vitamin C		244 (n)	76.0 (%)
Tocilizumab		15 (n)	4.7 (%)
Tocilizumab dose			
400 mg		10 (n)	66.7 (%)
800 mg		5 (n)	33.3 (%)
Cytokine adsorption		22 (n)	6.9 (%)
Mortality			
Overall mortality		66 (n)	20.6 (%)
14-day mortality		24 (n)	7.5 (%)
28-day mortality		55 (n)	17.1 (%)
Laboratory test variable (n: 321)	Mean ± SD	Median	Min-max
Glucose (mg/dL)	145.23 ± 64.21	123	68–418
AST (U/L)	41.39 ± 44.90	29	8–408
ALT (U/L)	37.43 ± 32.22		4–315
Creatinine (mg/dL)	1.04 ± 0.69		
Albumin (g/dL)	3.72 ± 0.51		
Leukocyte (10_3_/mm_3_)	6892.97 ± 3147.48		
Lymphocyte (10_3_/mm_3_)	1237.16 ± 670.93		
N/L	5.76 ± 6.54		
Platelet (10_3_/mm_3_)	209.97 ± 775.5		
Troponin (ng/L)	12.03 ± 22.16		
Myoglobin (ng/mL)	86.15 ± 195.52		
LDH (U/L)	317.22 ± 137.14		
Ferritin (μg/L)	404.18 ± 505.9		
CRP (mg/dL)	82.14 ± 85.28		
Procalcitonin (ng/mL)	0.98 ± 7.23		
IL-6 (pg/mL) (on admission)	106.28 ± 202.48		
IL-6 (pg/mL) (peak)	297.67 ± 826.66		
D-dimer (μg/L) (on admission)	717.74 ± 3719.13		
D-dimer (μg/L) (peak)	1817.3 ± 5886.06		

LMWH: Low-molecular-weight heparin, AST: Aspartate aminotransferase, ALT: Alanine aminotransferase, N/L: Neutrophil-to-lymphocyte ratio, LDH: Lactate dehydrogenase, CRP: C-reactive protein, IL-6: Interleukin 6

**Table 2:** Demographic and clinical characteristics by the treatment groups.

Variable	Group1 (n: 44)	Group 2 (n: 31)	Group 3 (n: 73)	Group 4 (n: 33)	Group 5 (n: 82)	Group 6 (n: 41)	Group 7 (n: 17)	*p* value	Overall (n: 321)
Age^a^ (years) (mean ± SD)	47.20 ± 13.58	48.97 ± 14.80	58.42 ± 13.73	54.39 ± 13.61	62.99 ± 14.25	56.41 ± 12.21	51.59 ± 12.98	<0.05^a^	56.11 ± 14.67
Sex male n(%)	21 (47.7)	11 (35.5)	48 (65.8)	24 (72.7)	47 (57.3)	27 (65.9)	11 (64.7)	>0.05	189 (58.9)
Smoker^b^ (n: 274) n (%)	5 (14.3)	7 (24.1)	15 (25.4)	8 (25.8)	31 (40.3)	13 (44.8)	8 (57.1)	<0.05^b^	87 (31.8)
Time from symptom onset to treatment (days) mean (min–max)	2 (0–14)	4 (0–24)	2 (0–10)	3 (0–11)	3 (0–15)	4 (0–12)	3 (0–7)	=0.651	3 (0–24)
Stay in ICU* (days) mean (min–max)	–	–	12 (1–82)	17 (7–35)	9.5 (2–28)	14 (6–65)	16 (6–40)	>0.05	12 (1–82)
Inpatient clinic stay^c^ (days) mean (min–max)	5 (0–18)	5 (0–16)	5 (0–21)	8 (0–33)	7 (0–30)	7 (0–20)	7 (0–17)	<0.05^c^	6 (0–33)
Total stay^d^ (days) mean (min–max)	5 (3–18)	5 (3–16)	8 (3–83)	11 (3–53)	10 (3–50)	13 (4–67)	20 (7–45)	<0.05^d^	9 (3–83)
Need for ICU n (%)	1 (2.3)	0 (0.0)	24 (32.9)	10 (30.3)	30 (36.6)	19 (46.3)	14 (82.4)	>0.05	98 (30.5)
Mechanical ventilation n(%)	1 (2.3)	0 (0.0)	18 (24.7)	7 (21.2)	19 (23.2)	11 (26.8)	11 (64.7)	>0.05	67 (20.9)
Pneumonia severity (mild) n (%)	26 (59.1)	18 (58.1)	26 (35.6)	9 (27.3)	14 (17.1)	9 (22.0)	0 (0.0)	>0.05	102 (31.8)
Pneumonia severity (moderate) n (%)	12 (27.3)	10 (32.3)	25 (34.2)	13 (39.4)	23 (28.0)	12 (29.3)	8 (47.1)	>0.05	103 (32.1)
Pneumonia severity (severe)	6 (13.6)	3 (9.7)	22 (30.1)	11 (33.3)	45 (54.9)	20 (48.8)	9 (52.9)	>0.05	116 (36.1)

a The mean age of the patients in Group 5 was significantly higher than all other groups. The mean age of the patients in Group 1 is significantly lower than those in Groups 3, 4, 5, and 6. The mean age of the patients in Group 2 is significantly lower than those in Groups 3, 5, and 6 (p < 0.05, one-way ANOVA, post hoc LSD test was used for statistical analysis).

b Smoking rate in Group 1 was significantly lower than in Group 7 (p < 0.05, chi-square, post hoc Bonferroni test was used for statistical analysis).

c The length of stay in inpatient clinic was significantly shorter in Group 2 than in Groups 4 and 5 (p < 0.05, the Kruskal–Wallis and post hoc pairwise comparisons test were used for statistical analysis).

d Total length of stay was significantly shorter in Groups 1 and 2 compared to other Groups and longer in Group 7 (p < 0.05, the Kruskal–Wallis and post hoc pairwise comparisons test were used for statistical analysis).

The variables are presented as mean ± SD or median (min–max) and n (%).

* Intensive care unit

**Table 3:** Laboratory data for the presenting values by the main treatment groups.

Variable	Group 1 (n: 44)	Group 2 (n: 31)	Group 3 (n: 73)	Group 4 (n: 33)	Group 5 (n: 82)	Group 6 (n: 41)	Group 7 (n: 17)	*p* value	Overall (n: 321)
Fasting blood glucose (mg/dL)	98 (76–245)	114 (81–221)	128 (68–394)	109 (85–238)	136 (81–418)	126.5 (88–300)	129 (83–224)	<0.05	123 (68–418)
AST (U/L)	23 (14–67)	25 (15–115)	31 (8–391)	30 (13–85)	23 (11–111)	35.5 (9–408)	29 (15–64)	<0.05	29 (8–408)
ALT (U/L)	24 (6–61)	19.5 (7–161)	32.5 (4–315)	29 (10–110)	29.5 (8–110)	30 (8–242)	40 (16–65)	<0.05	29 (4–315)
Creatinine (mg/dL)	0.8 (0–4)	0.7 (1–1)	0.9 (0–5)	0.9 (0–5)	1 (0–8)	1 (1–3)	0.9 (0–2)	<0.05	0.9 (0–8)
Albumin (g/dL)	4.3 (2.3–5.0)	4.2 (3.3–5.0)	3.6 (2.4–4.6)	3.8 (2.8–4.6)	3.6 (2.1–4.4)	3.6 (2.1–4.6)	3.6 (2.6–4.1)	<0.05	3.7 (2.1–5)
Leukocyte (10^3^/mm^3^)	5400 (2700–11600)	5200 (2800–12400)	5600 (3300–19400)	6000 (3800–16100)	6700 (770–17100)	7100 (2000–16100)	7000 (1200–17300)	<0.05	6100 (770–19400)
Lymphocyte (10^3^/mm^3^)	1350 (200–3000)	1400 (200–3800)	1200 (80–4500)	1000 (100–2200)	1100 (200–5400)	1000 (500–3000)	800 (300–1800)	<0.05	1200 (80–5400)
N/L	2.3 (1–13)	2 (1–8)	3.7 (1–50)	3.9 (2–57)	5 (1–32)	5.2 (1–23)	9 (2–37)	<0.05	3.8 (1–57)
Platelet (10^3^/mm^3^)	206.5 (21–405)	214 (90–323)	196 (28–385)	202 (74–363)	197 (79–661)	200 (82–379)	199 (85–517)	=0.895	202 (21–661)
Troponin (ng/L)	3 (3–18)	3 (3–21)	6.5 (3–165)	3 (3–104)	5 (2–176)	6.5 (3–34)	3 (3–150)	<0.05	4 (2–176)
Myoglobin (ng/mL)	22 (21–522)	25 (21–80)	57 (21–690)	38 (21–734)	50 (16–2621)	45 (21–445)	45 (20–120)	<0.05	38.5 (16–2621)
LDH (U/L)	208 (119–476)	229.5 (13–402)	300 (136–975)	272.5 (156–563)	311.5 (90–715)	334 (153–758)	340 (215–747)	<0.05	284 (13–975)
Ferritin (μg/L)	95 (3–1788)	90 (4–770)	223.5 (5–1967)	256 (26–2443)	303 (29–4279)	305.5 (21–1941)	454.5 (86–2525)	<0.05	244 (3–4279)
CRP (mg/dL)	10 (1–300)	13.5 (0–180)	55 (1–473)	40 (1–208)	91 (6–370)	88 (2–229)	87 (23–318)	<0.05	54 (0–473)
Procalcitonin (ng/mL)	0.06 (0–26)	0.06 (0–0)	0.08 (0–30)	0.1 (0–3)	0.08 (0–23)	0.1 (0–1)	0.6 (0–106)	<0.05	0.09 (0–106)
IL-6 (presenting) (pg/mL)	12 (2–1408)	6 (2–41)	89.5 (6–638)	45.5 (6–295)	35 (2–934)	67 (3–1309)	61 (2–508)	<0.05	42 (2–1408)
IL-6 (peak) (pg/mL)	22 (2–5000)	6.3 (2–41)	132.15 (0–638)	69 (17–5000)	60 (2–1128)	113 (3–5000)	135.5 (28–3054)	<0.05	83.5 (0–5000)
D-dimer (presenting) (μg/L)	183 (10–5207)	118 (36–794)	293 (70–62484)	198 (38–5428)	262 (48–5152)	272 (106–2978)	287 (105–16676)	<0.05	238.5 (10–62484)
D-dimer (peak) (μg/L)	204 (19–10900)	148 (36–1405)	515 (70–62484)	408 (43–6455)	497 (48–10540)	511 (156–11791)	1350 (150–55001)	<0.05	370 (19–62484)

- The variables are presented as mean ± SD or median (min–max) and n (%).

- AST: Aspartate aminotransferase, ALT: Alanine aminotransferase, N/L: Neutrophil-to-lymphocyte ratio, LDH: Lactate dehydrogenase, CRP: C-reactive protein, IL-6: Interleukin 6

**Table 4:** Comorbidities by treatment groups.

Variable n (%)	Group1 (n: 44)	Group 2 (n: 31)	Group 6 (n: 41)	Group 4 (n: 33)	Group 5 (n: 82)	Group 6 (n: 41)	Group 7 (n: 17)	Overall (n: 321)
Comorbidities a	13 (29.5)	14 (45.2)	41 (56.2)	13 (39.4)	53 (64.6)	25 (61.0)	8 (47.1)	167 (52.0)
DM b	4 (9.1)	9 (29.0)	30 (41.1)	5 (15.2)	30 (36.6)	11 (26.8)	2 (11.8)	91 (28.3)
HT c	6 (13.6)	7 (22.6)	26 (35.6)	8 (24.2)	35 (42.7)	18 (43.9)	4 (23.5)	104 (32.4)
CAD, HF, HL	4 (9.1)	4 (12.9)	12 (16.4)	1 (3.0)	16 (19.5)	5 (12.2)	1 (5.9)	43 (13.4)
Obesity	1 (2.3)	0 (0.0)	5 (6.8)	3 (9.1)	12 (14.6)	5 (12.2)	4 (23.5)	30 (9.3)
Other	4 (9.1)	4 (12.9)	21 (28.8)	8 (24.2)	20 (24.4)	13 (31.7)	4 (23.5)	74 (23.1)

a There was a significant difference between Groups 1 and 5 in terms of comorbid disease (p < 0.05, chi-square, post hoc Bonferroni test was used for statistical analysis).

b DM was less in Group 1 than in Groups 3 and 5 (p < 0.05, chi-square, post hoc Bonferroni test was used for statistical analysis).

c HT was less in Group 1 than in Groups 5 and 6 (p < 0.05, chi-square, post hoc Bonferroni test was used for statistical analysis).

-The variables are presented as n (%).

-Abbreviations: DM: Diabetes mellitus, HT: Hypertension, CAD: Coronary artery disease, HF: heart failure, HL: Hyperlipidemia

**Table 5:** Main treatment groups and mortality.

Variable n (%)	Group 1 (n: 44)	Group 2 (n: 31)	Group 3 (n: 73)	Group 4 (n: 33)	Group 5 (n: 82)	Group 6 (n: 41)	Group 7 (n: 17)	Overall (n: 321)	*p* value
Overall mortality	1 (2.3)	0 (0.0)	20 (27.4)	6 (18.2)	21 (25.6)	11 (26.8)	7 (41.2)	66 (20.6)	0.05
28-day mortality	1 (2.3)	0 (0.0)	18 (24.7)	4 (12.1)	20 (24.4)	6 (14.6)	6 (35.3)	55 (17.1)	0.05

The variables are presented as n (%).

**Table 6:** Treatments and practices other than main treatment by the groups.

Variable	G 1 (n: 44)	G 2 (n: 31)	G 3 (n: 73)	G 4 (n: 33)	G 5 (n: 82)	G 6 (n: 41)	G 7 (n: 17)	*p* value	Overall (n: 321)
Steroid a	0 (0.0)	0 (0.0)	29 (39.7)	6 (18.2)	57 (69.5)	19 (46.3)	13 (76.5)	<0.05^a^	124 (38.6)
Methylprednisolone (n: 124)	-	-	27 (93.1)	6 (100.0)	27 (47.4)	15 (78.9)	10 (76.9)	>0.05	85 (26.5)
Dexamethasone (n: 124) b	-	-	2 (6.9)	0 (0.0)	30 (52.6)	4 (21.1)	3 (23.1)	<0.05^b^	39 (31.5)
Time from symptom onset to the administration of steroids (days)	-	-	5(0–11)	9.5 (3–10)	6(0–15)	7 (0–15)	7 (1–10)	>0.05	6 (0–15)
Immune plasma c	0 (0.0)	0 (0.0)	12 (16.4)	5 (15.2)	18 (22.0)	9 (22.0)	14 (82.4)	<0.05^c^	58 (18.1)
Time from symptom onset to the administration of immune plasma (days)	-	-	9.5 (5–16)	11 (7–23)	8 (1–18)	10.5 (0–13)	6 (2–14)	>0.05	8 (0–23)
LMWH d	39 (88.6)	2 (6.5)	66 (90.4)	24 (72.7)	81 (98.8)	37 (90.2)	17 (10.0)	<0.05^d^	266 (82.9)
Time from symptom onset to the administration of LMWH e (days)	2 (0–14)	7 (5–9)	3 (0–11)	8 (3–21)	5 (0–15)	5 (0–11)	4 (1–10)	<0.05^e^	4 (0–21)
Tocilizumab f (n: 320)	0 (0.0)	0 (0.0)	4 (5.5)	1 (3.0)	1 (1.2)	6 (14.8)	3 (17.6)	<0.05^f^	15 (4.7)
Vitamin C g	22(50.0)	9 (29.0)	59 (80.8)	29 (87.9)	80 (97.6)	29 (70.7)	16 (94.1)	<0.05^g^	244(76.0)
Cytokine adsorption h	1 (2.3)	0 (0.0)	1 (1.4)	4 (12.1)	4 (4.9)	4 (9.8)	8 (47.1)	<0.05^h^	22 (6.9)

a Steroid usage was higher in Groups 5, 6, 7 than others (p < 0.05).

b Groups 3 and 5 were different from each other in terms of dexamethasone usage (p < 0.05).

c Group 7 differed from the others in the use of immune plasma (p < 0.05).

d Group 2 differed from the others in terms of LMWH use (p < 0.05).

e LMWH start time was higher in group 4 than in Groups 1, 3, 5; lower in Group 1 than in 5 and 6 (p < 0.05).

f Use of tocilizumab in Groups 5 and 7 was different from each other (p < 0.05).

g Use of vitamin C was higher in Groups 4, 5, and 7 than others (p < 0.05).

h Group 7 differed from Groups 1, 2, 3, 5, and 6 in terms of cytokine adsorption (p < 0.05).

- Chi-square, post hoc Bonferroni test was used for statistical analysis and the variables are presented as n (%).

- G: Group, LMWH: Low-molecular-weight heparin

**Table 7:** Survival by the demographic and clinical characteristics.

Variables	Current status		Total	*p* value
	Alive	Dead		
Age (years)	53.09 ± 14.11	67.77 ± 10.39	56.11 ± 14.67	<0.001
Sex				0.548
Male	148 (78.3)	41 (21.7)	189 (58.9)	
Female	107 (81.1)	25 (18.9)	132 (41.1)	
Smoker (n: 274)				
Yes	148 (79.1)	39 (20.9)	187 (68.2)	0.130
No	76 (87.4)	11 (12.6)	87 (31.8)	
Severity of pneumonia				
Mild	96 (94.1)	6 (5.9)	102 (31.8)	<0.001
Moderate	83 (80.6)	20 (19.4)	103 (32.1)	
Severe	76 (65.5)	40 (34.5)	116 (36.1)	
Need for Intensive care unit				
No	221 (99.1)	2 (0.9)	223 (69.5)	<0.001
Yes	34 (34.7)	64 (65.3)	98 (30.5)	
Mechanical ventilation				
No	246 (96.9)	8 (3.1)	254 (79.1)	<0.001
Yes	9 (13.4)	58 (86.6)	67 (20.9)	
Steroid type (n: 124)				
Methyl prednisolone	48 (56,.5)	37 (43.5)	85 (68.5)	0.380
Dexamethasone	26 (66.7)	13 (33.3)	39 (31.5)	
Immune plasma				
No	234 (89.0)	29 (11.0)	263 (81.9)	<0.001
Yes	21 (36.2)	37 (63.8)	58 (18.1)	
Tocilizumab (n: 320)				
No	253 (83.0)	52 (17.0)	305 (95.3)	<0.001
Yes	1 (6.7)	14 (93.3)	15 (4.7)	
Vitamin C				
No	65 (84.4)	12 (15.6)	77 (24.0)	0.259
Yes	190 (77.9)	54 (22.1)	244 (76.0)	
Cytokine adsorption				
No	247 (82.6)	52 (17.4)	299 (93.1)	<0.001
Yes	8 (36.4)	14 (63.6)	22 (6.9)	
Comorbidity				
No	136 (88.3)	18 (11.7)	154 (48.0)	<0.001
Yes	119 (71.3)	48 (28.7)	167 (52.0)	
Treatment group				
Group 1	43 (97.7)	1 (2.3)	44 (13.7)	0.001
Group 2	31 (100.0)	0 (0.0)	31 (9.7)	
Group 3	53 (72.6)	20 (27.4)	73 (22.7)	
Group 4	27 (81.8)	6 (18.2)	33 (10.3)	
Group 5	61 (74.4)	21 (25.6)	82 (25.5)	
Group 6	30 (73.2)	11 (26.8)	41 (12.8)	
Group 7	10 (58.8)	7 (41.2)	17 (5.3)	

The variables are presented as mean ± SD and n (%).

**Table 8:** Survival analyses by the treatment groups and main clinical characteristics.

	Mean survival (days)	STD ERR	95% CI lower limit	95% CI upper limit	*p* value
Overall survival	23.00	2.31	18.46	27.53	-
Sex					
Male	33.61	4.49	24.79	42.42	0.799
Female	31.25	4.02	23.36	39.13	
Smoker					
No	37.22	4.55	28.28	46.16	0.231
Yes	37.49	4.02	29.59	45.38	
Admission to ICU*					
No	32.64	0.25	32.14	33.14	<0.001
Yes	29.57	2.86	23.97	35.18	
Mechanical ventilation					
No	37.95	1.87	34.28	41.63	<0.001
Yes	25.11	2.55	20.11	30.11	
Severity of pneumonia					
Mild/Moderate	34.56	3.90	26.91	42.21	0.082
Severe	31.73	4.39	23.13	40.34	
Comorbidity					
No	30.36	3.07	24.33	36.39	0.349
Yes	31.90	3.49	25.04	38.75	
DM					
No	30.50	2.93	24.75	36.25	0.227
Yes	38.70	6.41	26.14	51.27	
HT					
No	36.18	4.97	26.43	45.93	0.151
Yes	29.47	3.51	22.59	36.36	
Obesity					
No	31.93	3.13	25.77	38.08	0.132
Yes	31.40	3.14	25.23	37.56	
Main treatment group					
Group 1	16.20	1.61	13.04	19.35	0.186
Group 2	-	-	-	-	
Group 3	25.02	4.66	15.87	34.16	
Group 4	38.80	5.52	27.97	49.63	
Group 5	26.99	3.30	20.53	33.46	
Group 6	33.32	6.32	21.09	45.53	
Group 7	33.69	4.08	25.68	41.70	

* ICU: Intensive care unit

**Table 9:** Logistic regression analysis of risk factors for mortality.

Variable	Multivariate analysis		
	OR	95% CI	*p* value
Age	1.074	1.021–1.129	0.005
Severity of pneumonia			
Mild	Ref	-	-
Moderate	2.448	0.448–13.378	0.302
Severe	3.622	0.784–16.743	0.099
Need for ICU*	14.780	2.522–86.613	0.003
Mechanical ventilation	29.177	7.849–108.450	<0.001
Immune plasma	1.093	0.326–3.662	0.886
Cytokine adsorption	1.240	0.280–5.495	0.777
Comorbidity	0.655	0.193–2.221	0.497

Nagelkerke R square: 0.805,

* ICU: Intensive care unit

## Footnotes

1 Republic of Turkey Ministry of Health (2020). COVID-19 (SARS-CoV-2 Infection) Guide, 11.03.2020 [online]. Website www.hsgm.saglik.gov.tr [accessed April 3, 2021].

2 Republic of Turkey Ministry of Health (2020). COVID-19 (SARS-CoV-2 Infection) Guide, 02.08.2020 [online]. Website https://toraks.org.tr/site/sf/nmf/2020/08/16c01709f0a799d5529e2f29648de7b4cc7dff09378 [accessed April 3, 2021].

3 Republic of Turkey Ministry of Health. COVID-19 (SARS-CoV-2 Infection) guide, 9.10. 2020. [online]. Website https://covid19.saglik.gov.tr/Eklenti/39061/0/covid19rehberieriskinhastatedavisipdf.pdf [accessed April 3, 2021].

4 T.C. Sağlık Bakanlığı COVID-19 immün (konvalesan) plazma tedarik ve klinik kullanim rehberi 2020: 1–21 [online]. Website https://shgm.saglik.gov.tr/Eklenti/39179/0/covid-19-immun-konvalesan-plazma-tedarik-ve-klinik-kullanim-rehberipdf.pdf [accessed April 6, 2021].
